# Co-trimoxazole-induced reproductive toxicity and placental-barrier disruption: impact on cell-cell junctions and ERK signaling pathway 

**DOI:** 10.3389/fcell.2026.1800328

**Published:** 2026-06-08

**Authors:** Mehrdad Azarmi, Hassan Saei, I. A. C. van Vugt, Suzan Thijssen, Belinda van ’t Land, Ruurd van Elburg, Johan Garssen, Gert Folkerts, Astrid Hogenkamp, Saskia Braber

**Affiliations:** 1 Division of Pharmacology, Utrecht Institute of Pharmaceutical Sciences (UIPS), Utrecht University, Utrecht, Netherlands; 2 Laboratory of Hereditary Kidney Disease, Inserm UMR 1163, Institute Imagine, Université Paris Cité, Paris, France; 3 Danone Research and Innovation, Utrecht, Netherlands; 4 Center for Translational Immunology, University Medical Center Utrecht, Utrecht, Netherlands; 5 Department of Pediatrics, Amsterdam University Medical Center, Emma Children’s Hospital, Amsterdam, Netherlands

**Keywords:** antibiotic, cell-cell junction, co-trimoxazole, *DUSP*, MAPK, placenta, pregnancy

## Abstract

Co-trimoxazole (CTX), a combination of sulfamethoxazole (SMZ) and trimethoprim (TMP), is used during pregnancies complicated by infections like Urinary Tract Infections (UTI) and Human Immunodeficiency Virus (HIV), despite potential fetal safety concerns. This study examines CTX’s effects on the placenta using both *in vivo* and *in vitro* models. Pregnant mice received CTX for 4 days during both early and late gestation. On gestational day 19, tissues were collected for analysis. Furthermore, *in vitro*, human BeWo placental cells were exposed to non-cytotoxic concentrations of CTX for 24 h. Gene expression was analyzed by bulk RNA sequencing with pathway analysis, and placental barrier function was assessed by measuring transepithelial electrical resistance (TEER) and fluorescein isothiocyanate-dextran (FITC-D) permeability. *In vivo*, CTX significantly reduced uterine weight and litter size (*p* < 0.05), indicating potential reproductive toxicity, which was supported by placental transcriptomic analysis. *In vitro*, bulk RNA-sequencing of CTX-treated BeWo cells exhibited differential expression of genes related to tight and adherens junctions, indicating impaired placental barrier integrity. This was accompanied by a significant reduction in TEER (*p* < 0.0001) and an increase in FITC-D permeability (*p* < 0.001). In addition, CTX activated the ERK1/2 (MAPK3) pathway, as indicated by increased ERK1/2 (*p* < 0.01) phosphorylation and downregulation of the ERK pathway negative regulators (*DUSP* genes). Additionally, *IL-6* expression levels were reduced in the amniotic fluid (*p* < 0.01) and placenta of CTX-treated mice, and a similar reduction was observed in CTX-treated BeWo cells at both the transcript (*p* < 0.05) and protein (*p* < 0.001) levels. In conclusion, CTX may induce reproductive toxicity and compromise placental barrier integrity, warranting further investigation into its safety during pregnancy.

## Graphical Abstract

**Figure:**
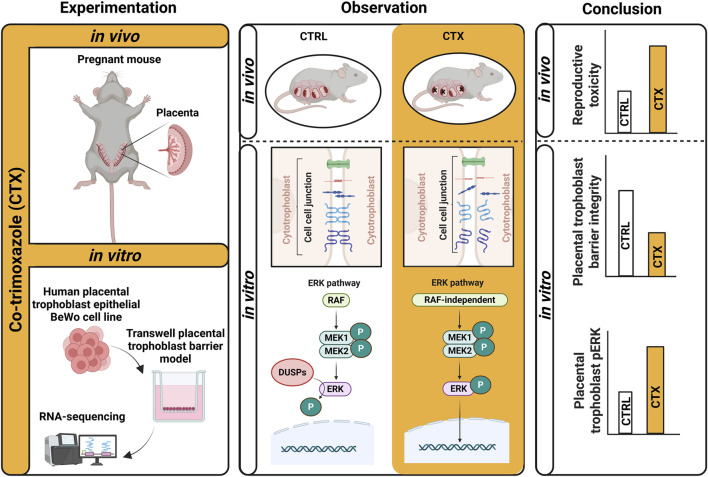
CTX, Co-trimoxazole; RNA, Ribonucleic Acid; CTRL, Control; ERK, Extracellular Signal-Regulated Kinase; MEK, Mitogen-Activated Protein Kinase Kinase; DUSP, Dual Specificity Phosphatase; P, Phosphorus; pERK, Phosphorylated-Extracellular Signal-Regulated Kinase.

## 1 Introduction

Antibiotics are a class of medicines widely prescribed to treat bacterial infectious diseases and have contributed greatly to increased life expectancy (Adedeji, 2016). There is an increase in research into the effects of antibiotic usage during pregnancy. This is an important area of research, as antibiotics comprise 80% of all medicines prescribed during pregnancy, and one in five pregnant individuals will use at least one antibiotic during gestation (Bookstaver et al., 2015; de Jonge et al., 2014). These antibiotics are often prescribed to treat or, in some high-risk populations, to prevent infections, such as urinary tract infections (UTI), respiratory tract infections, or sexually transmitted infections. If left untreated, these infections have detrimental effects on both maternal and fetal health, including spontaneous abortion (Bookstaver et al., 2015). A risk-benefit assessment should be conducted when prescribing antibiotics to pregnant individuals, as antibiotic exposure in pregnancy is linked to several adverse outcomes. Gestational antibiotic exposure is associated with an increased risk of preterm birth (Cantarutti et al., 2021; Nguyen et al., 2022), alterations in infant birth weight (Cantarutti et al., 2021) and a higher likelihood of childhood obesity (Mueller et al., 2015).

Co-trimoxazole (CTX), a combination of sulfamethoxazole (SMZ) and trimethoprim (TMP), is used to treat a range of infections during pregnancy, including gestational pneumonia and UTI, and is frequently recommended for HIV-positive pregnant individuals (Ford et al., 2014; Corrales et al., 2022; Betschart et al., 2020; Smilack, 1999; Muanda et al., 2018; MacLean, 2001). The global pooled prevalence of HIV infection among pregnant women is estimated at approximately 2.9%, highlighting the clinical relevance of CTX use during pregnancy (Wu et al., 2023). While CTX use in HIV-positive pregnant women is associated with reduced risk of preterm delivery and neonatal mortality (Walter et al., 2006), first-trimester CTX exposure results in a 2.94-fold increased odds of spontaneous abortion (Muanda et al., 2018). Therefore, its use during pregnancy is recommended only when maternal benefits outweigh potential fetal risks (Ford et al., 2014).

According to the Food and Drug Administration (FDA), CTX carries a category D pregnancy warning (Ford et al., 2014). Despite this, the World Health Organization (WHO) guidelines recommend CTX prophylactic use during all stages of pregnancy in HIV-infected women with a CD4 count ≤350 cells/mm^3^ (World Health Organization, 2014). Furthermore, UTI represents a major clinical concern during pregnancy and is among the most frequently diagnosed conditions, affecting approximately 23.9% of pregnancies (Salari et al., 2023). For gestational UTI, CTX is considered an effective antibiotic in selected cases throughout pregnancy when administered with folic acid supplementation (Corrales et al., 2022; Waltzer, 1981; Matuszkiewicz-Rowińska et al., 2015; Czajkowski et al., 2021).

CTX works as a bactericidal antibiotic through a two-step blockage of the bacterial folate metabolism, essential in the biosynthesis of nucleic acids and proteins. SMZ inhibits the bacterial synthesis of dihydrofolic acid by competing with para-aminobenzoic acid, while TMP selectively inhibits microbial dihydrofolate reductase, blocking the production of tetrahydrofolic acid from dihydrofolic acid (Ford et al., 2014; Walter et al., 2006; World Health Organization, 2014; Kemnic and Coleman, 2023). Although CTX is more selective for the bacterial reductase enzyme, it could also interfere with human folate metabolism (Ford et al., 2014).

Both SMZ and TMP can cross the placenta (Bawdon et al., 1991; van Hove et al., 2022; Prokopczyk et al., 1979), and reach the fetal circulation. Their pharmacokinetics in neonates differ from those in adults, with longer half-lives and reduced clearance rate due to immature organ function (Ryan et al., 2013), leading to prolonged exposure, which could pose a risk, as folate is essential in foetal development due to its importance in DNA synthesis. The antifolate abilities of CTX during the first trimester result in an increased risk of congenital malformations, including neural tube, cardiac, and urinary tract defects (Hernández-Díaz et al., 2000; Hernández-Díaz et al., 2001). However, reported rates of congenital abnormalities, low birth weight, spontaneous abortion, and neonatal death vary widely among studies, limiting the overall quality of the evidence (Ford et al., 2014). These potential adverse outcomes may result from disruptions in critical developmental processes during pregnancy, including impaired placental development and function (Thompson et al., 2024; Umapathy et al., 2024), which is pivotal for fetal growth and pregnancy maintenance.

The placenta, as the maternal-fetal interface, forms the sole link between the birthing parent and the fetus during pregnancy. It facilitates maternal-fetal substance exchange, hormone secretion, and barrier protection (Gude et al., 2004). The placental epithelium consists of undifferentiated cytotrophoblasts and differentiated syncytiotrophoblasts, which form the structural and biochemical barrier between the maternal and foetal blood, regulating placental and foetal development (Weiler, 2005). The integrity of the placental epithelial layer is maintained by junctional proteins, including tight junctions (TJs) and adherens junctions (AJs), which regulate the paracellular transport and the permeability of the placental epithelium (Gonzalez-Mariscal et al., 2003; Garcia‐Hernandez et al., 2017; Günzel and Yu, 2013; Tossetta et al., 2014; Marzioni et al., 2001). Any disruption to this junctional network can compromise the integrity of the epithelial layer and potentially affect foetal development (Tossetta et al., 2014). Increased epithelial permeability may enable toxins, pathogens, or other teratogenic compounds to cross the placental barrier; a tighter barrier could hinder the removal of foetal waste products, such as urate (Gude et al., 2004; Uehara et al., 2014).

To maintain this complex and dynamic placental environment, several molecular signaling pathways are involved, among which the mitogen-activated protein kinase (MAPK) pathway is particularly crucial (Nadeau and Charron, 2014). This pathway plays a central role in regulating trophoblast proliferation, differentiation, and survival, as well as mediating the placental response to various physiological and pathological stressors (Daoud et al., 2005; Charron et al., 2012; Zhou et al., 2023).

This study provides novel insights into the effects of CTX on placental barrier integrity and transcriptomic profiles. Despite its use during pregnancies complicated by HIV infection and UTI, the placental effects of CTX remain largely unexplored. To address this knowledge gap, we evaluated reproductive toxicity, CTX-induced changes in placental gene expression, and placental barrier integrity following CTX exposure using complementary *in vivo* murine pregnancy and *in vitro* placental barrier models.

## 2 Materials and methods

### 2.1 Compounds and reagents

CTX is a combination of SMZ and TMP in a fixed 5:1 ratio. Although administered in this ratio, both compounds behave as independent molecules *in vivo*, with respective half-lives of 10–13 h and 8–11 h. SMZ and TMP undergo hepatic metabolism and renal excretion as separate entities (COCKERILL and EDSON, 1991; Leegwater et al., 2025). For *in vivo* administration, CTX, consisting of SMZ and TMP in a 5:1 ratio, was prepared in PBS and administered orally to mice at a dose of 500 mg/kg/day.

For *in vitro* experiments, SMZ and TMP (Sigma-Aldrich, St. Louis, MO, United States) were dissolved in dimethyl sulfoxide (DMSO 0.1%) (Sigma-Aldrich) to prepare stock solutions of 20 mg/mL. The CTX stock solution was then prepared by mixing SMZ and TMP at a 5:1 ratio and diluted in antibiotic-free cell culture medium. Based on cytotoxicity assays (Figure 2), non-cytotoxic concentrations of SMZ (100 and 200 μg/mL), TMP (5 and 50 μg/mL), and CTX (5 and 50 μg/mL) were selected for subsequent experiments. CTX (50 μg/mL; 5:1 SMZ: TMP ratio) corresponds to ∼41.7 μg/mL SMZ and ∼8.3 μg/mL TMP. The SMZ component approximates human plasma levels (∼57 μg/mL), while TMP exceeds typical plasma levels (∼1.72 μg/mL) (U.S. Food and Drug Administration, 2021) but remains within high-dose therapeutic ranges (Brown, 2014). As CTX is the clinically co-prescribed formulation, these conditions reflect the most physiologically relevant exposure in BeWo trophoblast cells. Working dilutions were freshly prepared in antibiotic-free cell culture medium before each experiment. PD98059 (Invitrogen, Frederick, MD, United States) is a selective mitogen-activated protein kinase kinase 1 (MEK1) inhibitor commonly used to block the Extracellular signal-Regulated Kinase 1/2 (ERK1/2) signaling pathway. For ERK pathway inhibition in BeWo cells, PD98059 was applied at a final concentration of 50 µM for 24 h, as previously reported to be effective in cytotrophoblasts (Daoud et al., 2005; Chen et al., 2000).

### 2.2 Animals

Six-week-old female and nine-week-old male specific pathogen-free (SPF) C3H/HeOuJ mice were purchased from Charles River Laboratories and housed in the animal facility of Utrecht University at controlled temperature (21 °C ± 2 °C) and humidity (50%–55%), with a reversed 12:12 h light/dark cycle (lights on from 7 a.m. till 7 p.m.) with *ad libitum* access to food and tap water. All experimental procedures were carried out during the light/inactive part of the cycle, and all repeated measurements were performed at the same time block of the day. Animals were kept in individually ventilated cages (IVC) (22 cm × 16 cm×14 cm, floor area 350 cm^2^, Technilab-BMI, Someren, the Netherlands) and wood-chip bedding (Technilab- BMI), tissues (VWR, the Netherlands), and shelters were available as cage enrichment. The animals were provided standard diets (pelleted food, AIN-93G, Sniff Spezialdiäten, Soest, Germany) and routine care upon arrival at the animal facility, continuing until the end of the experiment. Upon arrival in the animal facility, mice were acclimatized for 1 week, after which the female mice were trained for oral gavage over the course of a week through daily administration of the gavage needle to minimize the gavage-related stress during pregnancy. This study was conducted in accordance with institutional guidelines for the care and use of laboratory animals established by the Animal Ethics Committee of Utrecht University. All animal procedures related to the purpose of the study were approved under license of the national competent authority (CCD), securing full compliance with the European Directive 2010/63/EU for the use of animals for scientific purposes, with the approval number of AVD10800202316818.

### 2.3 Breeding, pregnancy, and antibiotic exposure

Upon mice’s arrival at the animal facility, male and female mice were housed separately in different cages (2 females/cage and 3 males/cage). After a week of acclimatization, male breeders were housed solitarily for 5 days before being co-housed with their assigned pair of female mice. Forty-eight hours before mating (male-female co-housing), the cages of the male and female mice were exchanged to synchronize the females’ estrus cycles via the Whitten effect. Thereafter, the two female mice were transferred back to their original cage, including the male breeder. Male and female mice were co-housed for 4 days for breeding, with a ratio of one male to 2 females. Twenty-four hours post-breeding was considered as gestational day 0 (GD0). After 4 days of breeding, the breeder male mice were removed from the cages. The bred female mice received CTX via oral gavage at a volume of 400 μL, suspended in PBS (500 mg/kg/day) (Barnes et al., 2013; Sivalingam et al., 2008) during two distinct periods of pregnancy (GD1-GD6 and GD13-GD18). The control pregnant mice received 400 µL PBS orally during the mentioned treatment period. On GD19, the pregnant dams were euthanized under deep anesthesia using isoflurane inhalation, followed by decapitation, whereafter samples, including blood, placenta, and amniotic fluid, were isolated for *ex vivo* analysis.

### 2.4 Cell culture

The human placenta choriocarcinoma trophoblast BeWo cell line, obtained from the American Type Culture Collection (ATCC-CCL-98, Rockville, MD, United States; passages 42–46), was cultured in cell culture flasks (Corning, Kennebunk, ME, United States) using F-12K medium (Kaighn’s modification of Ham’s F-12 medium; Gibco, Thermo Fisher Scien tific, Paisley, United Kingdom), supplemented with 10% foetal calf serum (Science, Brebières, France), and a 1% mixture of penicillin (100 U/mL) and streptomycin (100 μg/mL). Cells were maintained in a humidified atmosphere of 5% CO_2_ at 37 °C, and the medium was refreshed three times a week until the cells reached confluence (7 ± 1 days). Confluent cultures were passaged by washing with PBS and detaching the cells using 0.05% trypsin (Gibco, Thermo Fisher Scientific, Wilmington, DE, United States) and 0.54 mM ethylene diamine tetra-acetic acid (EDTA). For exposure to antibiotics, BeWo cells were seeded in either 96 or 24-well plates (CellStar, Greiner Bio-One, Frickenhausen, Germany) at a cell density of 10,000 and 30,000 cells per well, respectively. Once confluency of ±80% was reached (7 ± 1 days), the cells were exposed to CTX (and SMZ, TMP) using penicillin-streptomycin-free medium for 24 h. All experiments contained control cells unexposed to any reagent.

### 2.5 Cytotoxicity assay

To evaluate the cytotoxic effects and determine safe concentrations of CTX on the BeWo cell line, a broad range of SMZ, TMP, and CTX concentrations was initially tested. The cytotoxic effects of these antibiotics were investigated by measuring Lactate Dehydrogenase (LDH) leakage from cells into the cell culture medium using the CytoTox 96® Non-Radioactive Cytotoxicity Assay Kit (Promega Corporation, Madison, WI, United States) in 96-well flat-bottom plates. Four antibiotic concentrations, including 100, 200, 300, 400 μg/mL for SMZ, 25, 50, 100, 200 μg/mL for TMP, and 25, 50, 100, 200 μg/mL for CTX, were investigated to determine the acute cytotoxicity effect (24 h exposure). The concentration range for 24 h exposure was selected from a broader preliminary range and based on estimated human serum levels (Dudley et al., 1984; Campioli et al., 2022). To investigate the cytotoxicity of chronic exposure to SMZ, TMP, or CTX, BeWo cells were treated for 5 days with the highest non-toxic concentrations determined from acute antibiotic exposure, followed by treatment with a range of lower concentrations. Every 24 h, 100 µL of the supernatant was gently removed and replaced with fresh medium containing the same antibiotic concentration. Considering the LDH instability during freeze-thaw cycles, LDH release was measured directly, according to the manufacturer’s instructions. The chronic cytotoxicity was calculated from the added LDH absorbance values of 5 days and using the formula (cytotoxicity (%) = (E/M) × 100), where E represents the experimental LDH release, and M represents the maximum LDH release. Maximum LDH release was determined by incubating positive control cells with a standard lysis buffer, Triton X-100. In addition, day-specific cytotoxicity was also calculated during chronic exposure (5 days). The LDH release was quantified by measuring absorbance at 490 nm using the GloMax Discover microplate reader (Promega Corporation).

### 2.6 RNA sequencing

#### 2.6.1 Bulk RNA sequencing

Bulk RNA sequencing was performed on BeWo cells following 24-h exposure to SMZ (200 μg/mL), TMP (50 μg/mL), or CTX (50 μg/mL), as well as a DMSO (0.1%) control and an untreated control. Total RNA was extracted using the SV Total RNA Isolation System (Promega Corporation), and the RNA quantity and integrity were analyzed with the Agilent Fragment analyzer. Furthermore, bulk RNA sequencing was also performed on mouse placental tissue collected from control and CTX-treated dams. Total RNA was isolated from placental samples by the sequencing provider according to standard proprietary protocols. RNA quantity and integrity were assessed by the provider before library preparation and sequencing. The mRNA library construction and paired-end sequencing were conducted with a DNA nanoball and polymerase-based stepwise sequencing system (DNBSEQ) in BGI (Shenzhen, China). The paired-end FASTQ files were checked for adaptor sequences and poor-quality bases and short read length with Fastp (v.0.23.2) using the following parameters (-q 20 –L 50) (Chen et al., 2018). Quality-assured trimmed reads were mapped to the GRCh38 assembly of the human genome using the splice-aware mapper Hisat2 (v.2.2.1) (Kim et al., 2015). The overall mappability in all samples was above 96 percent for ∼40 to 50 million 150bp reads. The raw count matrix was generated by feature Counts using GRCh38. p14 V^44^ human gene annotation file (Liao et al., 2014). Gene counts were normalized, and differential gene expression (DEG) analysis was performed with the DESeq2 package in R (v.4.3.1) (Love et al., 2014). The principal component analysis (PCA) was performed on VST-normalized gene count data for better interpretation of the expression-based similarities and variations across samples. A gene was considered as differentially expressed only if it was statistically significant (p-adj <0.05) and |log2| fold change was >0.5. This threshold was selected to retain genes with moderate but potentially biologically relevant changes in expression that would otherwise be excluded under more stringent criteria (e.g., |log2FC| ≥ 1). Heatmaps were generated on the DESeq2 normalized counts, and a z-score transformation was done before visualization.

#### 2.6.2 Over-representation and gene set enrichment analysis

Query DE gene lists obtained from different comparison groups were subjected to over-representation analysis using enrichR (Kuleshov et al., 2016), a web-based tool for gene set enrichment, to identify biological pathways and functional categories over-represented among the DEGs. The msigdbr R package (v.1.10.0) was used to retrieve functional gene sets and their member genes for different categories, including C2 for KEGG, Reactome, and C5 for Gene Ontology (MF; molecular function, CC; cellular component, and BP; biological processes) (Bhuva et al., 2023). Significantly represented pathways (*p*-adj <0.05) and gene ontologies were extracted and compared between different *in vitro* groups. The ClusterProfiler (v.4.8.3) package was used for the gene set enrichment analysis (GSEA) (Wu et al., 2021). Genes were ranked by a signed ranking metric (Log_2_FC), and gene ontology and pathway analysis were done with the following parameters (nPerm = 10,000, minGSSize = 3, maxGSSize = 800, *p*valuecutoff = 0.05, and *p*AdjustMethod = “BH”). Significantly enriched pathways (*p* < 0.05) were identified and further examined by Western blot and qPCR with respect to their biological relevance.

#### 2.6.3 Partial least squares-discriminant analysis of murine placental transcriptomes

Supervised multivariate analysis using Partial Least Squares-Discriminant Analysis (PLS-DA), and sparse PLS-DA was performed on transcriptome data from the *in vivo* experiment using the mixOmics package (Rohart et al., 2017) to identify latent components and features that discriminate between experimental groups (CTX-treated from untreated control). Variance-stabilized expression values obtained using the DESeq2 “vst” function were used as input for this analysis. Due to the small sample size (N = 4), the model showed overfitting under leave-one-out cross-validation, as indicated by a negative Q^2^. However, a negative Q^2^ does not invalidate the exploratory use of PLS-DA for feature prioritisation; it indicates that the model is not suitable for predictive classification. The model achieved good training-set discrimination (high R^2^Y = 69.2% and 25.1% across the two components) but lacked predictive generalisability. Accordingly, any features identified by this method should be considered exploratory and require validation in future studies with larger sample sizes and complementary molecular analysis.

### 2.7 TEER measurement and paracellular tracer flux assay

To investigate the effects of the SMZ, TMP, and CTX on the functioning of the *in vitro* placental barrier, transepithelial electrical resistance (TEER) measurements and a paracellular dye flux assay were performed. BeWo cells were seeded at a cell density of 30,000 live cells per insert onto CoStar polyester membrane Transwell 6.5 mm inserts with 0.4 µm pores (Corning, Kennebunk) in a 24-well plate. Once the cells reached a confluent monolayer (10 ± 1 days), they were exposed to SMZ, TMP, or CTX for 24 h from apical and basolateral compartments. The integrity of the monolayer was assessed by measuring TEER at time points 0 h and 24 h post-exposure using the Millicell ERS-2 Volt-Ohm meter (Millipore, Temecula, CA, United States). Permeability of the BeWo cell monolayer was evaluated by adding 100 μg/mL of membrane-permeable 4 kDa fluorescein isothiocyanate-dextran (FITC-D, Sigma-Aldrich) to the apical compartment only. After 4 h of incubation, 50 µL of basolateral medium was collected, and fluorescence intensity was measured with a GloMax Discover microplate reader (Promega Corporation) at excitation and emission wavelengths of 485 and 520 nm, respectively. For the FITC-D permeability assay, background fluorescence was determined using cell culture medium without FITC-D (blank control), and this value was subtracted from all experimental samples to correct for baseline signal.

### 2.8 RNA isolation and qPCR

To assess mRNA expression, BeWo cells were seeded in 24-well transwell plates as described above and exposed for 24 h to SMZ, TMP, or CTX. Cells were lysed in 175 µL of RNA lysis buffer containing β-mercaptoethanol, and total RNA was extracted using the SV Total RNA Isolation System (Promega Corporation). For *in vivo* analyses, mouse placentas were collected on gestational day 19, snap-frozen in liquid nitrogen, and stored at −80 °C until processing. Placental tissues were homogenized in the same lysis buffer using a Precellys® 24 homogenizer (Bertin Technologies, France), and RNA isolation was performed following the manufacturer’s protocol. The RNA content was determined using the Nanodrop One/One^C^ Spectrophotometer (Thermo Fisher Scientific, Madison, WI, United States). The purity of RNA samples was assessed using the 260/280 nm and 260/230 nm absorbance ratios, with ratios above 2 considered indicative of high purity. Reverse transcription of the mRNA samples into cDNA was conducted using the iScript cDNA synthesis kit (Bio-Rad Laboratories, Hercules, CA, United States) and the T100 Thermal Cycler (Bio-Rad Laboratories). Quantification of cDNA samples was performed using SYBR Green qPCR. The reaction mixtures were prepared in a 96-well hard-shell PCR plate (Bio-Rad Laboratories), and 1 µL of the appropriate cDNA sample was added to each well. Amplifications were performed using the CFX96 Touch™ Real-Time PCR Detection System (Bio-Rad Laboratories). The selected primers were commercially synthesized (Biolegio, Nijmegen, Netherlands), and mRNA expression levels were measured relative to the expression of the housekeeping gene *β-actin* and *RPL13A* (Supplementary Table S1).

### 2.9 Protein isolation and western blot

Western blot analysis was performed to assess the activation of Extracellular Signal-Regulated Kinase (ERK) signaling in both human BeWo placental cells and mouse placental tissue. For *in vitro* cell analysis, BeWo cells were seeded in 24-well plates, as previously described, and exposed to SMZ, TMP, or CTX for 24 h. Cells were lysed with ice-cold Pierce™ RIPA buffer (Thermo Scientific, Rockford, IL, United States) containing protease inhibitors (Roche Diagnostics, Mannheim, Germany) and phosphatase inhibitors (Thermo Scientific), followed by incubation on ice for 30 min. For *in vivo* analysis, mouse placentas were collected on gestational day 19, snap-frozen in liquid nitrogen, and stored at −80 °C. Tissues were homogenized in the same lysis buffer using a Precellys® 24 tissue homogenizer (Bertin Technologies) and processed under identical conditions. For both models, lysates were centrifuged at 14,000 × g for 15 min at 4 °C, and supernatants were collected. Total protein concentrations were determined using the Pierce™ BCA protein assay kit (Thermo Scientific). Equal amounts of denatured protein were separated by Sodium Dodecyl Sulfate-Polyacrylamide Gel Electrophoresis (SDS-PAGE) using Mini-PROTEAN TGX and Criterion™ TGX Gels (4%–20%, Bio-Rad Laboratories) and transferred onto Polyvinylidene fluoride (PVDF) membranes using the Trans-Blot Turbo Transfer System (Bio-Rad Laboratories). Membranes were blocked for 1 h at room temperature in blocking buffer (0.1% (v/v) Tween-20 in PBS and 5% (w/v) milk proteins), then incubated overnight at 4 °C with primary antibodies against ERK1/2 (4695, Cell Signaling Technology, Danvers, MA, United States) and phosphorylated ERK1/2 (9101, Cell Signaling Technology) diluted in blocking buffer. After washing with PBS-T, membranes were incubated with HRP-conjugated secondary antibodies (Dako, Glostrup, Denmark) at a 1:3,000 dilution for 1 h at room temperature. Chemiluminescent detection was performed using Clarity™ Western Enhanced Chemiluminescence (ECL) Substrate (Bio-Rad Laboratories), and signals were captured using the ChemiDoc™ MP Imaging System (Bio-Rad Laboratories). Band intensity was quantified using ImageJ software (v.1.54G), and normalization was performed using Glyceraldehyde-3-phosphate dehydrogenase (GAPDH) as a loading control (monoclonal mouse anti-human GAPDH, 60004-1-Ig, Proteintech, Manchester, United Kingdom). Protein expression levels were expressed as fold change (FC) relative to the control group.

### 2.10 Enzyme-linked immunosorbent assay

Interleukin-6 (IL-6) protein levels were quantified in both *in vitro* and *in vivo* samples using enzyme-linked immunosorbent assay (ELISA). For *in vitro* analysis, BeWo cells were initially seeded at 30,000 cells per well and exposed to SMZ, TMP, or CTX for 24 h. Following exposure, the culture supernatants were collected and analyzed for IL-6 concentration using a commercial ELISA kit (Invitrogen, San Diego, CA, United States), using the manufacturer’s protocol. For *in vivo* analysis, IL-6 concentrations in amniotic fluid collected from CTX-treated and control mice were measured using a murine ELISA kit, following the manufacturer’s instructions (Promega Corporation). For ELISAs performed on *in vitro* and *in vivo* samples, 100 μL of each sample was used without further dilution. The absorbance at 450 nm was measured using a GloMax Discover microplate reader (Promega Corporation), and concentrations were calculated based on standard curves prepared from the kit-provided standards.

### 2.11 Statistics

Statistical analysis was performed using GraphPad Prism version 10.4.1 (627). Data normality was assessed by the Shapiro-Wilk test. Comparisons among multiple groups were conducted using one-way Analysis of Variance (ANOVA), followed by a Bonferroni multiple-comparison *post hoc* test. Comparisons between two independent groups were performed using an unpaired two-tailed Student’s t-test. All results are shown as mean ± Standard Error of the Mean (SEM), and differences were considered statistically significant at *p* < 0.05 (*p < 0.05, **p < 0.01, ***p < 0.001, ****p < 0.0001). RNA-sequencing related statistical analyses were performed using R software, as already described in the corresponding methods section.

## 3 Results

### 3.1 Co-trimoxazole induces reproductive toxicity in a murine pregnancy model

**FIGURE 1:**
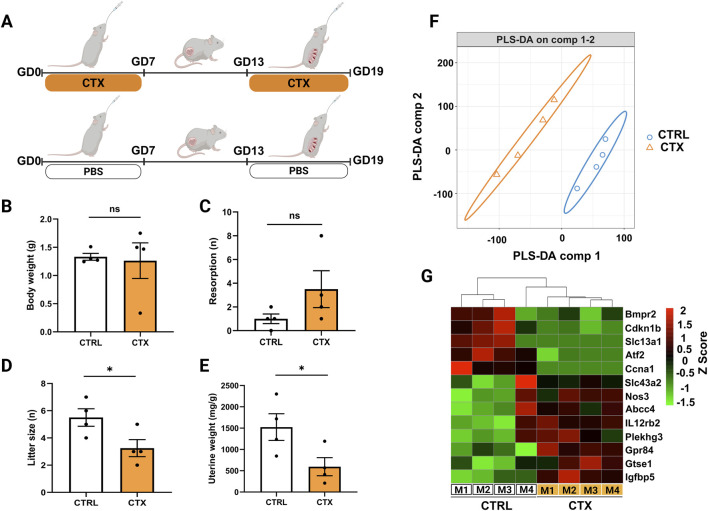
Cotrimoxazole (CTX) exposure during pregnancy reduces litter size and uterine weight and alters placental gene expression in mice. **(A)** Experimental design of *in vivo* CTX treatment during the first and last 6 days of pregnancy in a 19-day gestational period **(B)** Body weight changes in control and CTX-treated pregnant mice. Body weight was calculated by subtracting the weight on GD19 from GD0 and normalized to the number of embryos. **(C)** Number of resorbed embryos in control and CTX-treated pregnant mice. Resorbed embryos were counted after euthanizing the pregnant dams on GD19. **(D)** Number of embryos (litter size) in control and CTX-treated pregnant mice. **(E)** Uterine weight relative to body weight and the number of embryos in control and CTX-treated pregnant mice. **(F)** PLS-DA score plot showing clear separation between the control and CTX groups along components 1 and 2. Supervised clustering revealed distinct group-specific patterns, enabling improved discrimination for downstream analysis. **(G)** Heatmap of genes selected as exploratory candidates involved in reproductive function (shortlisted based on PLS-DA loading vectors and not validated), showing distinct expression patterns between control and CTX groups. The color scale represents z-score normalized expression values. Data were analyzed by Student’s t-test for graphs B-E and are presented as mean ± SEM. Both the CTRL and CTX groups include four mice (N = 4). Statistical significance is indicated as *p < 0.05, and ns represents non-significant differences between groups. GD, Gestational day; CTX, Co-trimoxazole; PBS, Phosphate-buffered saline; CTRL, Control; ns, Non-significant; PLS-DA, Partial Least Squares-Discriminant Analysis; Z-score, Indicates the deviation of a data point from the mean of a data set; A positive Z-score indicates that a data point is above the mean; a Z-score of 0 signifies that it equals the mean; and a negative Z-score indicates that it is below the mean; M1, Mouse 1; M2, Mouse 2; M3, Mouse 3; M4, Mouse 4.

Figure 1A illustrates the experimental procedure of CTX exposure to pregnant mice. Pregnant mice received CTX via oral gavage during the first (GD1-GD6) and last 6 days (GD13-GD18) of pregnancy. On GD19, the effects of CTX on gestational body weight, litter size, fetal resorption, and uterine weight were analyzed. As shown in Figure 1B, the body weight remained unaffected in CTX-treated pregnant mice compared to control mice. While the increase in observed resorption in CTX-treated pregnant mice was not statistically significant (Figure 1C), these mice exhibited a significantly reduced litter size compared to the control group (*p* < 0.05) (Figure 1D). Furthermore, exposure to CTX significantly reduced the uterine weight (*p* < 0.05) compared to control pregnant mice (Figure 1E). To further investigate the possible molecular mechanisms underlying the observed effect of CTX on reproductive outcomes, transcriptomic profiling of murine placental tissue was conducted. Initial data from bulk RNA-sequencing of placenta samples, based on an unsupervised PCA, revealed substantial overlap between the control and CTX groups, indicating limited discriminatory power, suggesting that DEGs could not be clearly identified (Supplementary Figure S1). Given that the control and CTX groups were not distinctly segregated along the principal components, PLS-DA, a supervised multivariate method to enhance class separation, was applied. PLS-DA distinguished between control and CTX groups with clear clustering along the first two components, which represent the main axes of variation used for group separation (Figure 1F). A subset of genes contributing to the separation between control and CTX groups was identified via PLS-DA. Among these, thirteen genes with reported roles in placental function and reproductive toxicity were highlighted based on literature evidence. As depicted in Figure 1G, these genes showed altered expression in CTX-treated mice compared to control mice. The expression of *Slc43a2, Nos3, Abcc4, IL12rb2, Plekhg3, Gpr84, Gtse1,* and *Igfbp5* was increased in CTX-treated mice compared to control mice, while *Bmpr2, Cdkn1b, Slc13a1, Atf2,* and *Ccna1* expression was decreased. This exploratory transcriptomic analysis identified CTX-associated placental gene expression patterns that may contribute to altered reproductive outcomes, rather than establishing definitive mechanistic causality.

### 3.2 Co-trimoxazole induces cytotoxic effects in BeWo cells

**FIGURE 2:**
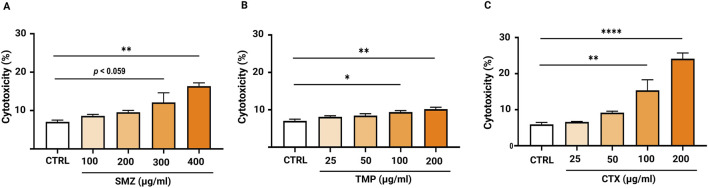
Lactate dehydrogenase (LDH) leakage from BeWo cells after 24 h exposure to increasing concentrations of SMZ, TMP, or CTX. Cytotoxicity (%) of **(A)** SMZ, **(B)** TMP, and **(C)** CTX in BeWo cells. Cytotoxicity was assessed by measuring LDH leakage. All graphs represent three independent experiments (N = 3), each including three technical repeats (n = 3). Data were analyzed by one-way ANOVA and are presented as mean ± SEM. Statistical significance is indicated as: *p < 0.05, **p < 0.01, ****p < 0.0001. CTRL: control, SMZ: Sulfamethoxazole, TMP: Trimethoprim, CTX: Co-trimoxazole.

The cytotoxicity of CTX, SMZ, and TMP in the human placental epithelial cell line (BeWo) was determined *in vitro* by measuring lactate dehydrogenase (LDH) leakage into the cell culture supernatant, as an indicator of compromised cell membrane integrity. As shown in Figure 2A, a significant increase in LDH leakage was observed with rising concentrations of SMZ, indicating cytotoxicity at 400 μg/mL (*p* < 0.01) after 24 h of exposure. Furthermore, increasing concentration of TMP led to a significant rise in LDH leakage, with 100 μg/mL (*p* < 0.05) and 200 μg/mL (*p* < 0.01) identified as cytotoxic (Figure 2B). CTX concentrations of 100 μg/mL (*p* < 0.01) and 200 μg/mL (*p* < 0.0001) were determined to be significantly cytotoxic after 24 h exposure compared to unexposed control cells (Figure 2C). SMZ (200 μg/mL), TMP (50 μg/mL), and CTX (50 μg/mL) were selected as non-toxic concentrations for the remaining experiments. Notably, CTX and SMZ showed cytotoxic effects even at lower concentrations after chronic exposure (5 days) to the BeWo cell line (Supplementary Figure S2).

### 3.3 Co-trimoxazole induces differential gene expression in BeWo cells

**FIGURE 3:**
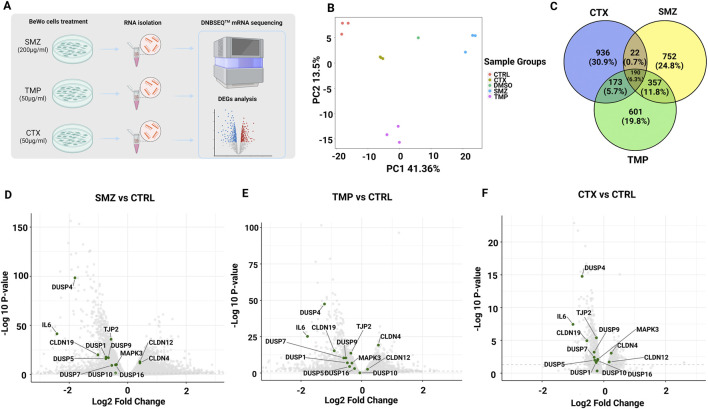
Transcriptomic analysis of BeWo cells after SMZ, TMP, or CTX exposure for 24 h. **(A)** Schematic of the experimental design for bulk RNA sequencing. **(B)** PCA of untreated BeWo cells and BeWo cells treated with SMZ (200 μg/mL), TMP (50 μg/mL), CTX (50 μg/mL), or DMSO (0.1%). **(C)** Venn diagram illustrating the distribution and overlap of DEGs among CTX- (blue), SMZ- (yellow), and TMP- (green) treated cells. Each circle represents the set of DEGs identified for one treatment, with overlapping regions indicating shared genes among the CTX-, SMZ-, and TMP-treated cells. Numbers and percentages represent the count and proportion of DEGs in each section relative to the total. **(D–F)** Volcano plots displaying differential gene expression in CTX-, SMZ, and TMP-treated cells, respectively, compared to untreated control cells. Each point represents a gene, plotted by log2 fold change (x-axis) and log10 adjusted *p*-value (y-axis). Genes with high statistical significance and large expression changes are located toward the top and sides of the plot. Labeled genes were selected based on their biological relevance to the ERK and cell-cell junction pathways. These pathways were identified as significantly enriched by GSEA, which relies on pathway-level enrichment rather than individual gene significance. Consequently, some labeled genes may not independently meet predefined DEG cutoff thresholds. DEGs were statistically analyzed using DESeq2, with significance defined as *p* < 0.05, using three independent experiments (N = 3), each including three pooled technical repeats (n = 3). CTRL, control; SMZ, Sulfamethoxazole; TMP, Trimethoprim; CTX, Co-trimoxazole; RNA, Ribonucleic Acid; mRNA, Messenger Ribonucleic Acid; DNBSEQ, DNA Nanoball Sequencing technology; DEG, Differentially Expressed Genes; DMSO, dimethyl sulfoxide; PC1, first principal component; PC2, second principal component.

Total RNA was isolated from BeWo cells exposed to CTX (50 μg/mL), SMZ (200 μg/mL), and TMP (50 μg/mL) for 24 h. Subsequently, bulk RNA-sequencing was conducted using DNBSEQ technology for genome-wide gene expression analysis (Figure 3A). As shown in Figure 3B, PCA revealed distinct segregation among untreated control cells, cells exposed to SMZ, TMP, CTX, or DMSO (vehicle control), occupying different regions in the PCA plot. This was shown by the first principal component (PC1) and second principal component (PC2), which accounted for 41.36% and 13.5% of the variation, respectively, indicating differential gene expression profiles. As depicted in Figure 3C, a Venn diagram was generated to compare the sets of DEGs across SMZ, TMP, or CTX treatments. The analysis revealed that 190 genes (6.3%) were altered, not only by CTX exposure, but also by CTX’s individual components, SMZ and TMP. However, CTX uniquely affected 936 genes (30.9%), while 752 genes (24.8%) were affected by SMZ, and 601 genes (19.8%) by TMP. Figures 3D–F shows volcano plots comparing each antibiotic treatment to untreated controls, revealing biologically relevant genes to the ERK pathway and cell-cell junction complex, including *MAPK3* and several dual-specificity phosphatases (*DUSPs*), key negative regulators of the ERK/MAPK signaling pathway (De Roo et al., 2025), as well as TJ-related genes. In SMZ-treated BeWo cells (Figure 3D), *IL-6* was strongly downregulated, along with MAPK-related genes such as *DUSP1, DUSP4, DUSP5, DUSP7,* and *DUSP9*, as well as TJ-related genes like *claudin-19* (*CLDN19*). TMP treatment (Figure 3E) elicited a similar pattern with consistent downregulation of *IL-6, DUSP4, DUSP5, DUSP7*, *DUSP9,* and *CLDN19*. In CTX-treated cells (Figure 3F), *IL-6, DUSP4, and CLDN19* were also notably downregulated compared to the untreated control cells. Together, these findings indicate a common modulation of IL-6, MAPK signaling, and TJ-related genes across all treatments.

### 3.4 Co-trimoxazole alters the cell-cell junctional network and disrupts barrier integrity in BeWo cells

**FIGURE 4:**
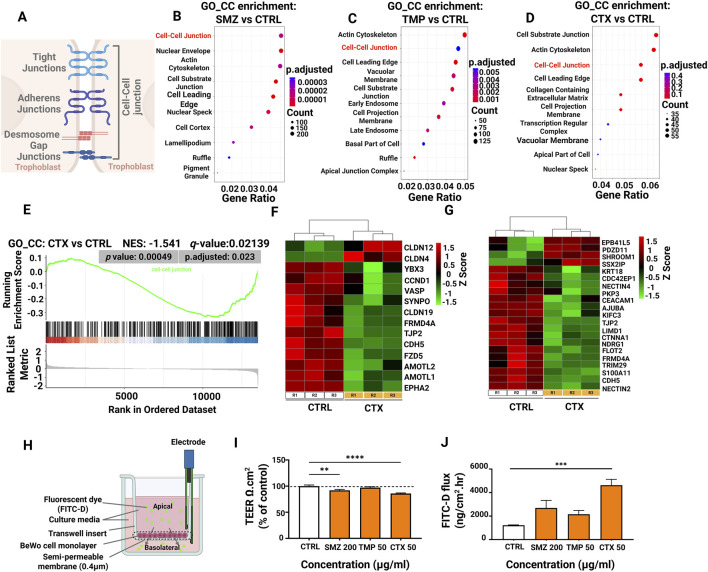
The effect of SMZ, TMP, or CTX on the cell-cell junctional network and barrier integrity in BeWo cells. **(A)** Composition of the cellular junctional network, including tight and adherens junctions, within the epithelial layer of placental trophoblast cells. **(B–D)** Dot plots of ORA results indicating the top enriched gene sets associated with GO Cellular Component terms in SMZ- (200 μg/mL) **(B)** TMP- (50 μg/mL) **(C)** and CTX- (50 μg/mL) **(D)** treated BeWo cells. **(E)** GSEA plot showing the enrichment of the cell-cell junction in CTX-treated BeWo cell line with an NES of −1.541. **(F)** Curated heatmap of tight junction genes from the enriched cell-cell junction gene set in untreated (control) and CTX-treated BeWo cells. **(G)** Curated Heatmap of adherens junction genes from the enriched cell-cell junction gene set in untreated (control) and CTX-treated BeWo cells. **(H)** Schematic illustration of the experimental setup using a transwell insert model. **(I)** Bar graph depicting transepithelial electrical resistance (TEER) as a percentage of the control (untreated BeWo cells) for SMZ- (200 μg/mL), TMP- (50 μg/mL), and CTX- (50 μg/mL) treated cells. **(J)** Permeability of FITC-D through the BeWo cell monolayer (expressed as FITC-D flux) after treatment with SMZ (200 µ g/mL), TMP (50 µ g/mL), and CTX (50 µ g/mL). All graphs represent three independent experiments (N = 3), each including three technical repeats (n = 3) (technical repeats were pooled for downstream RNA-sequencing analysis). Data were analyzed by one-way ANOVA and are presented as mean ± SEM. Statistical significance is indicated as: **p < 0.01, ***p < 0.001, and ****p < 0.0001. GO_CC, Gene Ontology Cellular Component; SMZ, Sulfamethoxazole; CTRL, Control; TMP, Trimethoprim; CTX, Co-trimoxazole; Count represents the number of the involved genes in each GO cellular component gene set; Gene Ratio, represents the percentage of the genes that significantly correlated with the enriched cellular component gene sets. NES, Normalized Enrichment Score; *q*-value, adjusted *p*-value controlling for the False Discovery Rate (FDR); Z-score, Indicates the deviation of a data point from the mean of a data set; A positive Z-score indicates that a data point is above the mean; a Z-score of 0 signifies that it equals the mean, and a negative Z-score indicates that it is below the mean. R1, Biological repeat 1; R2, Biological repeat 2; R3, Biological repeat 3.

Cell-cell junctions, especially TJs and AJs, connect neighboring cells to the cytoskeleton, forming a cohesive network throughout placental tissue (Leach et al., 2000) (Figure 4A). Exposure of BeWo cells to CTX, or its individual components SMZ or TMP, for 24 h altered the gene expression associated with the junctional complex, resulting in enrichment of the cell-cell junction gene set among the top pathways in Over Representation Analysis (ORA) of Gene Ontology Cellular component (GO-CC) database (Figures 4B–D). GSEA analysis using GO-CC datasets revealed that the cell-cell junction gene set was significantly enriched only in CTX-treated BeWo cells (Figure 4E). The Normalized Enrichment Score (NES) of −1.541 indicates that the gene set members were predominantly positioned at the bottom of the ranked list, reflecting their downregulation in CTX-treated cells compared to control. A distinct expression pattern was observed between CTX-treated and control cells, with widespread downregulation of TJ and AJ-related genes in CTX-treated cells, including *CLDN19*. However, a small subset of TJ and AJ-related genes was notably upregulated in the CTX-treated cells compared to control cells, such as *CLDN4* and *CLDN12* (Figures 4F,G). To evaluate the functional impact of CTX, as well as individual treatments with SMZ and TMP, on the BeWo cell monolayer barrier, TEER and FITC-D permeability were assessed using a Transwell insert model (Figure 4H). The mean TEER value for the untreated confluent BeWo monolayer was 70.4 ± 3.19 Ω.cm^2^ and was defined as 100% in Figure 4I. As depicted in Figure 4I, CTX significantly decreased TEER values (*p* < 0.0001), followed by SMZ, which also caused a significant but less pronounced reduction after 24 h of incubation (*p* < 0.01). In contrast, TMP did not significantly affect TEER values. Consequently, CTX exposure resulted in a significant (*p* < 0.001) increase in paracellular transport of FITC-D across the BeWo monolayer from the apical to the basolateral compartment. However, after exposure to SMZ and TMP, the paracellular transport of FITC-D did not change significantly (Figure 4J).

### 3.5 Co-trimoxazole induces downregulation of *DUSPs* and activation of the ERK pathway (MAPK1/3) in BeWo cells

**FIGURE 5:**
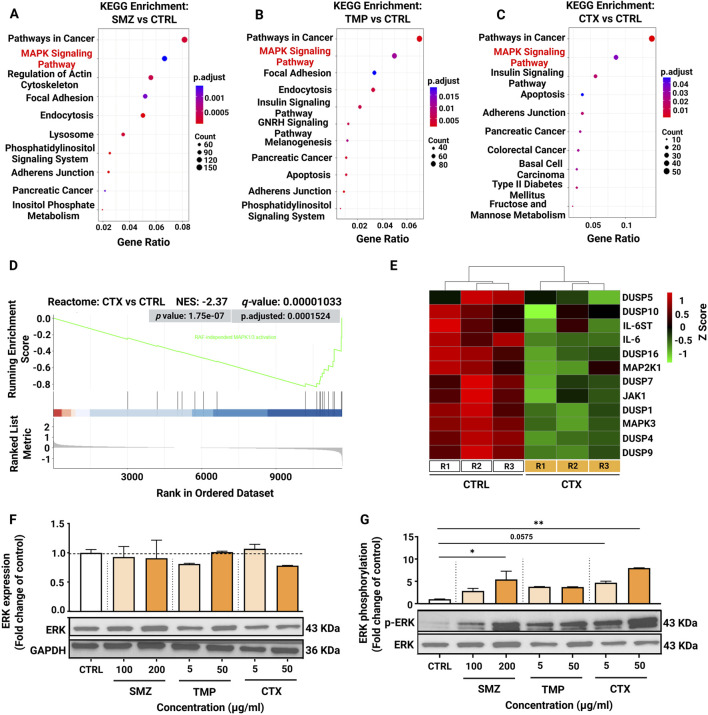
The impact of SMZ, TMP, or CTX on the ERK pathway in BeWo cells. **(A–C)** Dot plots indicating the top enriched KEGG pathways in SMZ- **(A)** TMP- **(B)** and CTX-treated **(C)** BeWo cells **(D)** GSEA enrichment plot highlighting the Raf-independent MAPK1/3 activation in CTX-treated BeWo cells, with an NES of 2.3. **(E)** Curated heatmap of Raf-independent MAPK1/3 activation pathway genes in CTX-treated cells. **(F)** Western blot of MAPK3 (ERK1) protein expression in SMZ, TMP, and CTX-treated cells relative to GAPDH as a reference protein. **(G)** Western blot of MAPK3 (ERK1) protein phosphorylation (p-ERK) in SMZ, TMP, or CTX-treated cells relative to ERK as reference protein. All graphs represent three independent experiments (N = 3), each including three pooled technical repeats (n = 3). Data were analyzed by one-way ANOVA and are presented as mean ± SEM. Statistical significance is indicated as: *p < 0.05, **p < 0.01. SMZ, Sulfamethoxazole; CTRL, Control; TMP, Trimethoprim; CTX, Co-trimoxazole; Count represents the number of the involved genes in each GO cellular component gene sets, Gene Ratio, represents the percentage of the genes that significantly correlated with the enriched cellular component gene sets. NES, Normalized Enrichment Score, *q*-value, adjusted *p*-value based on False Discovery Rate (FDR). RAF, Rapidly Accelerated Fibrosarcoma. A family of protein kinases (A-RAF, B-RAF, C-RAF) that are critical for the MAPK pathway. Z-score, Indicate the variance of data points from the mean of the data set. A positive z-score suggests that a data point is higher than the mean, a z-score of 0 signifies that the data point equals the mean, and a negative z-score indicates that the data point is lower than the mean. Reactome, a curated database offering insight into complex cellular interactions and processes, serving as a comprehensive resource for understanding various biological processes, KEGG, Kyoto Encyclopedia of Genes and Genomes; is a database resource that helps understand the biological system’s functions and utilities, from molecular-level information, including data from genome sequencing and high-throughput experimental technologies; ERK, Extracellular signal Regulated Kinase; p-ERK, phosphate- Extracellular signal Regulated Kinase; GAPDH, Glyceraldehyde-3-phosphate dehydrogenase; R1, Biological repeat 1; R2, Biological repeat 2; R3, Biological repeat 3; KDa, Kilodalton; TEER, Transepithelial electrical resistance; FITC-D, fluorescein isothiocyanate-dextran.

As shown in Figures 5A–C, exposure of BeWo cells to SMZ, TMP, or CTX induced differential expression of MAPK-related genes, resulting in the enrichment of the MAPK signaling pathway among the top pathways in ORA analysis of DEGs using the KEGG database. Although GSEA analysis showed that treatment of BeWo cells with SMZ and TMP alone did not affect the MAPK signaling pathway, CTX significantly (*p.*adjusted <0.0001) induced differential expression of involved genes in the Raf-independent MAPK1/3 (ERK2/1) pathway. The NES of 2.3 shows the downregulation of Raf-independent MAPK1/3 (ERK2/1 pathway) genes upon CTX exposure (Figure 5D). The curated heatmap of genes affected by CTX in the Raf-independent MAPK1/3 activation pathway gene set indicated downregulation of enriched genes, including *MAPK3*/*ERK1* and *MAP2K1,* an ERK-specific activator via dual-specific kinase activity (Figure 5E). Furthermore, *DUSPs*, such as *DUSP1*, *DUSP4*, *DUSP5*, *DUSP7*, and *DUSP9,* were also downregulated following CTX exposure. Moreover, IL-6 signal transducer (*IL-6ST*) and *Janus kinase 1* (*JAK1*) were downregulated in CTX-treated cells compared to control, all of which play a pivotal role in ERK activation. As depicted in Figure 5F, although SMZ, TMP, or CTX did not significantly affect the protein expression of *MAPK1/3* (*ERK2/1)* in BeWo cells, SMZ (*p* < 0.05) and CTX (*p* < 0.01) increased MAPK1/3 (ERK2/1) phosphorylation level, indicating activation of the ERK pathway (Figure 5G). The ERK-activating effect of CTX observed *in vitro* in BeWo cells was not recapitulated *in vivo* in mouse placental tissue; CTX exposure did not cause a significant change in ERK phosphorylation relative to controls (Supplementary Figure S3).

### 3.6 Co-trimoxazole suppresses *IL-6* expression in BeWo cells and in murine placental tissue

**FIGURE 6:**
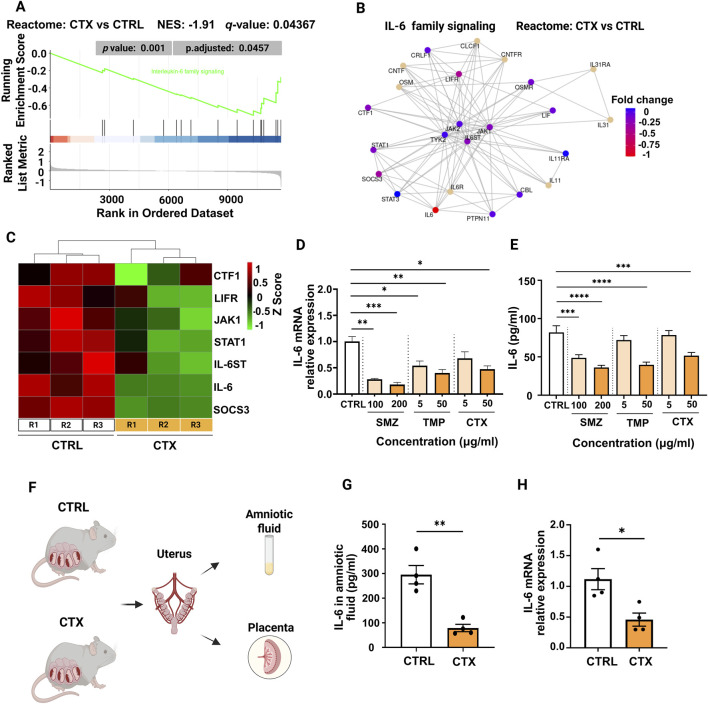
Analysis of IL-6 signaling and expression after CTX exposure in placental cells and tissue. **(A)** GSEA enrichment plot comparing the IL-6 signaling pathway between untreated and CTX-treated BeWo cells, using the Reactome database. **(B)** Gene network interactions within the IL-6 family signaling pathway. Each circle represents a gene within the IL-6 pathway, with color indicating its fold change in expression (red, downregulated; blue, upregulated). The connector lines indicate the connections and relationships among the genes. Genes included in the ranked list for GSEA analysis exhibit fold-change (FC) values ranging from −1 to 0, whereas genes not included in the ranked list are shown in yellow. **(C)** Curated heatmap of genes from the enriched IL-6 signaling pathway identified by GSEA analysis. The color bar represents gene expression changes, with green indicating low expression and red indicating high expression. **(D)** *IL-6* mRNA expression analysis via qPCR in SMZ-, TMP-, or CTX-treated and untreated (control) BeWo cells. **(E)** IL-6 protein analysis via ELISA in SMZ-, TMP-, and CTX-treated and untreated BeWo cells. **(F)** Schematic representation of *in vivo* samples from control and CTX-treated pregnant mice, including amniotic fluid and placenta, for IL-6 measurement. **(G)** IL-6 protein analysis via ELISA in amniotic fluid of control and CTX-treated pregnant mice and **(H)** *IL-6* mRNA expression analysis via qPCR in the placenta of control and CTX-treated pregnant mice. Graphs A-E represent three independent experiments (N = 3), each including three pooled technical repeats (n = 3). Graphs G and H represent four mice for each experimental group (N = 4). Data were analyzed by one-way ANOVA **(D,E)** or Student’s t-test **(G,H)** and are presented as mean ± SEM. Data are shown based on mean ± SEM. Statistical significance is indicated as: *p < 0.05, **p < 0.01, ***p < 0.001, ****p < 0.0001. CTX, co-trimoxazole; SMZ, Sulfamethoxazole; TMP, Trimethoprim; NES, Normalized Enrichment Score; *q*-value, adjusted *p*-value; False Discovery Rate (FDR); Z-score, Indicate the variance of a data point from the mean of the data set. A positive z-score suggests that a data point is higher than the mean, a z-score of 0 signifies that the data point equals the mean, and a negative z-score indicates that the data point is lower than the mean. R1, Biological repeat 1; R2, Biological repeat 2; R3, Biological repeat 3.

The progression of pregnancy and placental development depends on trophoblast cell activity, which is partially coordinated by cytokines and growth factors, including IL-6. GSEA analysis indicated that treatment of BeWo cells with SMZ and TMP alone did not affect the IL-6 signaling pathway, whereas CTX exposure resulted in a significant enrichment of IL-6 pathway-related genes (*q*-value = 0.04367) with a NES of −1.91 (Figure 6A). The negative NES indicates that IL-6 pathway genes are predominantly downregulated in the CTX-treated cells, suggesting that CTX suppresses IL-6 signaling activity in BeWo cells. Figure 6B shows the gene-gene interaction network of the IL-6 signaling pathway, illustrating changes in IL-6 family signaling induced by CTX compared to control. The greatest fold change was observed for *IL-6* itself, which appears in deep red among all genes in the network. The curated heatmap of IL-6 signaling-related genes altered by CTX exposure is shown in Figure 6C. The heatmap illustrates components of the IL-6/gp130 cytokine network, including key elements of the canonical IL-6 pathway (*IL-6, IL6ST, JAK1, STAT1, SOCS3*) and related IL-6 family members (*CTF1* and *LIFR*). As depicted in Figures 6D,E, not only CTX but also SMZ and TMP diminished the *IL-6* expression in BeWo cells at both the transcript and protein levels. To assess the translatability of CTX’s effect on IL-6 at the maternal-fetal interface, IL-6 protein levels in amniotic fluid and *IL-6* gene expression in placental tissue were quantified in pregnant mice treated with CTX during pregnancy (Figure 6F). As depicted in Figures 6G,H, CTX exposure during pregnancy significantly decreased the IL-6 protein (*p* < 0.01) and mRNA (*p* < 0.05) expression in amniotic fluid and placenta, respectively.

### 3.7 Co-trimoxazole suppresses *IL-6* expression in BeWo cells independently from ERK signaling

**FIGURE 7:**
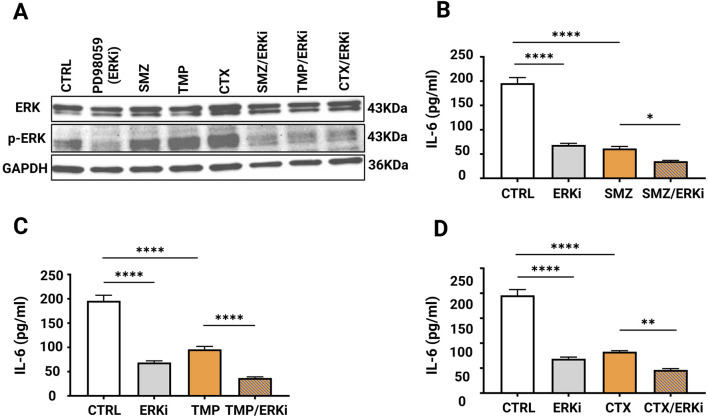
The effect of ERK inhibition on IL-6 production in BeWo cells exposed to SMZ, TMP, or CTX. **(A)** Representative Western blot showing total ERK and phosphorylated ERK (p-ERK) protein levels in BeWo cells treated with SMZ, TMP, CTX, or the ERK inhibitor PD98059 (ERKi), either alone or in combination (24 h). GAPDH was used as a loading control. **(B–D)** Quantification of IL-6 in BeWo cell culture supernatants by ELISA following treatment with SMZ, TMP, CTX, or the ERK inhibitor PD98059 (24 h), either alone or in combination. All graphs represent three independent experiments (N = 3), each including three pooled technical repeats (n = 3). Data were analyzed by one-way ANOVA and are presented as mean ± SEM. Statistical significance is indicated as: *p < 0.05, **p < 0.01, ****p < 0.0001. CTRL: Control ERK: Extracellular Signal-Regulated Kinases pERK: Phosphorylated Extracellular Signal-Regulated Kinases GAPDH: Glyceraldehyde 3-phosphate dehydrogenase ERKi (PD98059): a selective inhibitor of mitogen-activated protein kinase 1 (MEK1) that blocks extracellular signal-regulated kinase 1/2 (ERK1/2) signaling, SMZ, Sulfamethoxazole (200 μg/mL) TMP, Trimethoprim (50 μg/mL) CTX, Co-trimoxazole (50 μg/mL); SMZ/ERKi, combination of SMZ (200 μg/mL) and PD98059 (50 µM); TMP/ERKi, combination of TMP (50 μg/mL) and PD98059 (50 µM); CTX/ERKi, combination of CTX (50 μg/mL) and PD98059 (50 µM); IL-6, Interleukin-6.

To investigate the role of the ERK pathway in regulating IL-6 levels following SMZ, TMP, or CTX exposure in BeWo cells, we assessed ERK protein phosphorylation and IL-6 production with or without the ERK-specific inhibitor PD98059 (ERKi). Western blot analysis showed that SMZ, TMP, and CTX increased ERK phosphorylation (p-ERK) compared with control cells, which was effectively inhibited by ERKi (Figure 7A). ELISA analysis demonstrated that all treatments significantly (*p* < 0.0001) reduced IL-6 levels compared with controls (Figures 7B–D). Importantly, inhibition of ERK signaling did not restore IL-6 levels; instead, co-treatment with ERKi further reduced IL-6 levels.

## 4 Discussion

Clinical guidelines advise that women who meet criteria for CTX prophylaxis should continue therapy throughout pregnancy, as the risk of life-threatening infections outweighs the potential risk of fetal malformations (Ford et al., 2014; Organization, 2021). CTX, the co-formulated antibiotic of TMP and SMZ, remains clinically indicated in certain high-risk pregnancy situations, including gestational UTI, as well as an HIV-positive pregnancy (Ford et al., 2014; Corrales et al., 2022; Betschart et al., 2020).

Despite longstanding concerns, such as congenital malformation and maternal folic acid deficiency that might be followed by neonatal complications like neural tube defects and urinary tract defects, oral clefts (Smilack, 1999), the existing evidence does not justify completely avoiding CTX during pregnancy.

Although both TMP and SMZ cross the placenta, reaching fetal concentrations of 70%–100% of maternal levels (Ford et al., 2014), there is not sufficient research examining how these antibiotics might impact placental tissue and function, underscoring the need for further studies in this area. Therefore, in this study, we investigated the effects of CTX and its components, TMP and SMZ, on the placenta using an *in vivo* murine pregnancy model and a BeWo cell-based *in vitro* placental epithelium model.

CTX exposure during pregnancy significantly reduced litter size and uterine weight, with increased resorption (Figure 1), while maternal body weight remained unaffected. These outcomes are likely indicative of impaired implantation and/or suboptimal placental development, as CTX exposure was associated with alterations in placental gene expression related to cell cycle regulation, nutrient transport, and vascularization (Winterhager and Gellhaus, 2017; Jansson et al., 2006; Huang et al., 2021; Torry et al., 2007).

The defects observed with CTX in the current study align with previously reported effects of SMZ and CTX on fetal growth and ovarian development (L et al., 2022; Reel et al., 1992; Saberi et al., 2017). Animal models have also reported that antibiotic use during pregnancy, including β-lactams, macrolides, and norfloxacin, can induce reproductive toxicity (Nathanson et al., 2000; Karabulut et al., 2008; Aboubakr et al., 2014). In addition, gestational exposure to other antibiotics, like amoxicillin and metronidazole, has been associated with reduced fetal survival and impaired fetal growth, including increased fetal resorption and decreased litter size (Dai et al., 2024; AbdRabou et al., 2023; Huang et al., 2025).

Within this framework, the placenta represents a critical and frequently examined target due to its central role in supporting fetal growth and mediating maternal-fetal exchange (Jansson, 2016). Alterations in placental structure or gene expression can contribute to adverse pregnancy outcomes, highlighting the importance of investigating how gestational antibiotic exposure affects the placenta.

To investigate the mechanism of CTX-induced reproductive toxicity, placental samples from CTX-treated and control mice were analyzed using transcriptomics with a supervised approach. A total of thirteen genes, potentially involved in the reproductive toxicity observed in CTX-treated mice, showed altered expression levels in the placenta compared to controls. Among these, *Bmpr2, Cdkn1b, Slc13a1, Atf2,* and *Ccna1* were downregulated, whereas *Slc43a2, Nos3, Abcc4, IL12rb2, Plekhg3, Gpr84, Gtse1,* and *Igfbp5* were upregulated. These genes are biologically relevant to the placental function and are mechanistically linked to CTX-induced reproductive toxicity. They likely play pivotal roles in supporting fetal growth through diverse functions, including amino acid transport, placental vascularization, toxin efflux, protection against xenobiotics, immune regulation, junctional stability, and trophoblast invasion and differentiation. Modulation of these genes’ expression indicates that CTX exposure may impair key regulators of placental development and the maintenance of maternal-fetal homeostasis (Venkataraman and Kuo, 2005; Schulze et al., 2022; Xie et al., 2021; Liu et al., 2019; Han et al., 1996; Deng et al., 2024; Owaydhah et al., 2021; Krause et al., 2011; Joshi et al., 2016; Zourbas et al., 2001).

Given the alterations in placental gene expression related to reproductive toxicity observed in our *in vivo* murine pregnancy model, the human BeWo trophoblast cell line was employed as an *in vitro* model (Seyed Toutounchi et al., 2019) to examine the effects of CTX on the placental barrier.

The placental barrier is crucial for protecting the fetus by acting as a physical barrier against harmful agents (Kirkpatrick et al., 2025). Although antibiotics are widely studied for their effects on intestinal barrier integrity (Feng et al., 2019; Ran et al., 2020; Chen et al., 2021; Tulstrup et al., 2015; Bourke et al., 2019), evidence regarding their impact, especially that of CTX, on placental barrier integrity remains limited.

Transcriptomic analysis of BeWo cells after CTX exposure revealed that CTX impaired the placental trophoblast barrier integrity *in vitro*, as demonstrated by altered expression of TJ and AJ genes, including *CLDN19*, which play pivotal roles in maintaining placental integrity (Leach et al., 2000; Miranda et al., 2019; Pidoux et al., 2010). Although some TJ and AJ genes were upregulated following CTX exposure, most were downregulated.

This mixed expression profile suggests barrier dysregulation rather than coordinated junctional maintenance. The increased expression of certain junction-related genes (*CLDN4* and *CLDN12*) may represent a compensatory or stress-related attempt to maintain placental barrier integrity, whereas the reduced expression of key structural components such as *CLDN19* is likely to compromise the placental barrier. Consistent with this molecular profile, CTX exposure decreased TEER values and increased FITC-D permeability across BeWo cell monolayers, confirming a functional disruption of placental barrier integrity. While the adverse effects of antibiotics, including CTX, are commonly attributed to microbiome-mediated mechanisms, our findings demonstrate that CTX can also directly disrupt placental trophoblast barrier function in a microbiome-free *in vitro* model (Garcia et al., 2021; De Goffau et al., 2019; Panzer et al., 2023).

Besides CTX’s critical effects on cell-cell junctions in BeWo monolayers, CTX also activated the MAPK/ERK signaling pathway, as indicated by increased ERK1/2 phosphorylation. Proper placental barrier formation requires tightly regulated ERK/MAPK pathway activity, which is critical for normal growth, differentiation, and morphogenesis of placental cells (Nadeau and Charron, 2014; Daoud et al., 2005; Charron et al., 2012). CTX-induced activation of the ERK/MAPK pathway may be attributed to its suppressive impact on *DUSP* family gene expression in BeWo cells, particularly *DUSP5*, *DUSP7*, and *DUSP9*, which normally dephosphorylate and inactivate ERK to maintain appropriate pathway activity (Buffet et al., 2017; Kidger et al., 2017; Urness et al., 2008). Although other *DUSPs* suppressed by CTX may also contribute to ERK dephosphorylation (Tubita et al., 2025), their precise roles remain to be determined.

While our study is the first to demonstrate CTX-induced activation of the ERK pathway in BeWo placental trophoblast cells, other antibiotics, like colistin and macrolides, have been shown to modulate ERK signaling in other cell types, including bronchial epithelial cells and macrophages (Shinkai et al., 2006; Wang et al., 2019).

The observed disruptive effect of CTX on placental trophoblast barrier integrity may be associated with changes in ERK signaling, which has been implicated in the regulation of TJ and AJ gene expression (Mullin et al., 2005; Lipschutz et al., 2005; González-Mariscal et al., 2008; Cong and Kong, 2020). Furthermore, proper ERK signaling is required for normal placental growth, differentiation, and morphogenesis (Nadeau and Charron, 2014). ERK1/2 activation can disrupt the bronchial epithelial barrier by downregulating *E-cadherin*, *Z O -1*, *Occludin*, and *CLDN5* ​ (Zhou et al., 2024). Furthermore, ERK1/2-STAT3 pathway activation reduces TJ protein expression, lowers TEER, and increases permeability in nasal epithelial cells (Wu et al., 2024) with similar barrier-disruptive effects observed in the alveolar epithelium (Ruan et al., 2022).

Although CTX activated ERK signaling in BeWo cells *in vitro*, this effect was not observed in placental tissue *in vivo*. This difference likely results from compensatory regulatory mechanisms, cellular complexity, and differences in drug exposure between simplified *in vitro* conditions and the *in vivo* maternal-placental environment.

It is worth noting that ERK can also be strongly activated by pro-inflammatory cytokines (Djouad et al., 2009). In the BeWo cell model, CTX suppressed IL-6 signaling, significantly reducing *IL-6* expression at both the transcript and protein levels, while TNFα and IL-8 remained unaffected (Supplementary Figure S4). Furthermore, CTX exposure during pregnancy diminished the IL-6 levels in both amniotic fluid and placenta from *in vivo* murine pregnancy model.

IL-6 is a pleiotropic cytokine abundantly produced by placental trophoblasts and decidual cells at the maternal-fetal interface, where it plays an important role in placental function and immune regulation during pregnancy (Jovanović and Vićovac, 2009). IL-6 supports placental homeostasis by promoting trophoblast proliferation, migration, and invasion (Vilotić et al., 2022). A controlled pro-inflammatory environment during pregnancy is essential for successful implantation and placental development (Griffith et al., 2017). While our study demonstrated a local maternal-fetal suppressive effect of CTX on *IL-6* expression, a mouse pregnancy study with broad-spectrum antibiotics reported reduced systemic IL-6 production by splenocytes upon lipopolysaccharide stimulation (Benner et al., 2021).

Furthermore, clarithromycin reduced IL-6 concentrations in amniotic fluid during intra-amniotic infection, similar to the effect of CTX in our study, although this effect may be secondary to reduced bacterial burden or the immunomodulatory properties of the antibiotic (Kacerovsky et al., 2020). The ERK and IL-6 signaling pathways share a bidirectional relationship, where IL-6 activates the ERK pathway to promote proliferation, inflammation-induced ERK activation can, in turn, enhance IL-6 production (Leonard et al., 1999; Kitanaka et al., 2019). In our study, CTX activated ERK signaling *in vitro* without elevating IL-6 levels. Moreover, co-treatment of BeWo cells with an ERK-specific inhibitor and CTX further suppressed IL-6 release, indicating that CTX modulates the IL-6 pathway independently of ERK signaling, suggesting that alternative pathways, such as NF-κB signaling, may underlie the observed reduction in IL-6 (Liu et al., 2017). As previously mentioned, CTX is a fixed-dose combination of SMZ and TMP. Although each component can be prescribed individually, particularly TMP for uncomplicated UTI, the combination remains the most commonly used and clinically effective formulation due to its synergistic antibacterial activity.

Considering this clinical relevance, the present study primarily focused on the combined effect of CTX, especially in *vivo* murine experiments. Nevertheless, in the *in vitro* assays, both CTX and its individual components, SMZ and TMP, were evaluated to allow for direct comparison of their effects.

All three compounds, CTX, SMZ, and TMP, exhibited cytotoxicity toward placental trophoblast BeWo cells. However, CTX induced stronger cytotoxic effects at lower concentrations compared with either component alone. Transcriptomic analysis further revealed that CTX altered a larger proportion of DEGs (30.9%) than SMZ (24.8%) or TMP (19.8%). ORA indicated enrichment of cell-cell junction-related pathways across all treatments, while GSEA identified this effect exclusively in CTX-exposed BeWo cells. Functionally, CTX was the only compound that significantly disrupted trophoblast barrier integrity in the transwell model. Moreover, although SMZ, TMP, and CTX increased ERK phosphorylation, a significant activation of ERK was observed only in CTX-treated cells. Notably, all three compounds significantly reduced *IL-6* expression in BeWo cells. Collectively, these findings indicate that the combined formulation of CTX exerts a more pronounced and multifaceted disruptive effect on placental trophoblast function than either SMZ or TMP alone.

Despite providing novel insights into the gestational effects of CTX on placental function using both *in vitro* and *in vivo* models, this study has certain limitations that should be addressed. The BeWo cell model used in this manuscript is an accepted and employed placental trophoblast barrier model (Seyed Toutounchi et al., 2019; Poulsen et al., 2009; Fein et al., 2023), however, future studies using primary placental trophoblasts may provide greater physiological relevance. In addition, the *in vivo* experiments were conducted with a relatively small sample size (N = 4 per group), which may reduce statistical robustness and generalizability. In addition, placental genes associated with reproductive toxicity were identified using supervised PLS-DA based clustering for exploratory purposes only and require further validation by targeted approaches such as qPCR or WB.

It is also important to note that the microbiome, which was not examined in the current study, could provide additional insight into potential microbial influences on CTX-induced placental alterations. Another limitation is that the ERK signaling appears to contribute to the effects of CTX on trophoblast barrier integrity *in vitro*, but direct functional assessment of this pathway in BeWo cells, such as evaluating the impact of ERK inhibition on TEER value and TJs expression, was not performed. Such analyses could have provided deeper mechanistic insight into ERK-mediated regulation of placental barrier function that should be considered in future experiments. CTX-induced alterations in cell-cell junctions were detectable *in vitro* but not consistently observed in placental tissue *in vivo*, likely due to sample size or the greater cellular complexity and regulatory mechanisms present in the maternal-placental environment.

Taken together, our findings demonstrate that, despite CTX’s clinical benefits in treating maternal infections, CTX may pose potential risks during pregnancy by affecting both reproductive outcomes and placental health. In a murine model, CTX induced reproductive toxicity, while *in vitro,* CTX compromised placental barrier integrity, accompanied by activation of the ERK signaling pathway. Additionally, CTX suppressed IL-6 signaling within the placental environment, an effect that appeared to occur independently of ERK activation. These findings warrant heightened awareness regarding CTX use during pregnancy and support continued research into its placenta-specific molecular targets.

## Data Availability

The datasets presented in this study can be found in online repositories. The names of the repository/repositories and accession number(s) can be found in the article/Supplementary Material.
