# Supplementary material for: Co-trimoxazole-induced reproductive toxicity and placental-barrier disruption: impact on cell-cell junctions and ERK signaling pathway
Source: Front Cell Dev Biol. 2026 Jun 8;14:1800328. doi: 10.3389/fcell.2026.1800328 (PMC13284519; doi:10.3389/fcell.2026.1800328)
Supplement: Supplementary file 1 [file DataSheet1.docx]

Supplementary Material

**Supplementary Figures**

**
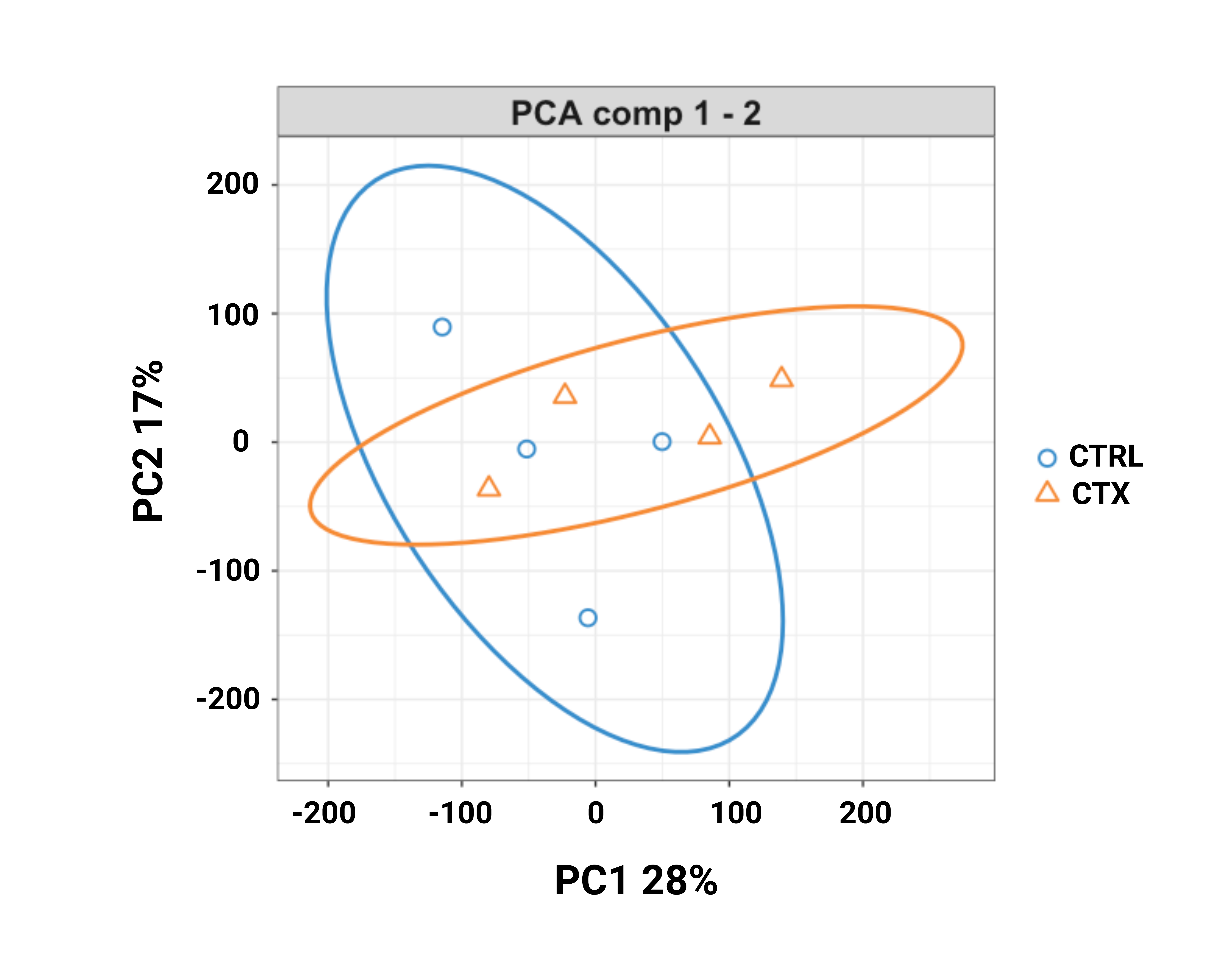
**

**Supplementary Figure 1.** PCA analysis of control (CTRL) and cotrimoxazole (CTX)-treated groups. PCA score plot based on the first two principal components (PC1 and PC2), explaining 28% and 17% of the total variance, respectively. **PCA**: Principal Component Analysis, **PC1:** Principal component 1, **PC2:** Principal component 2. **CTRL**: Control, **CTX:** Co-trimoxazole.


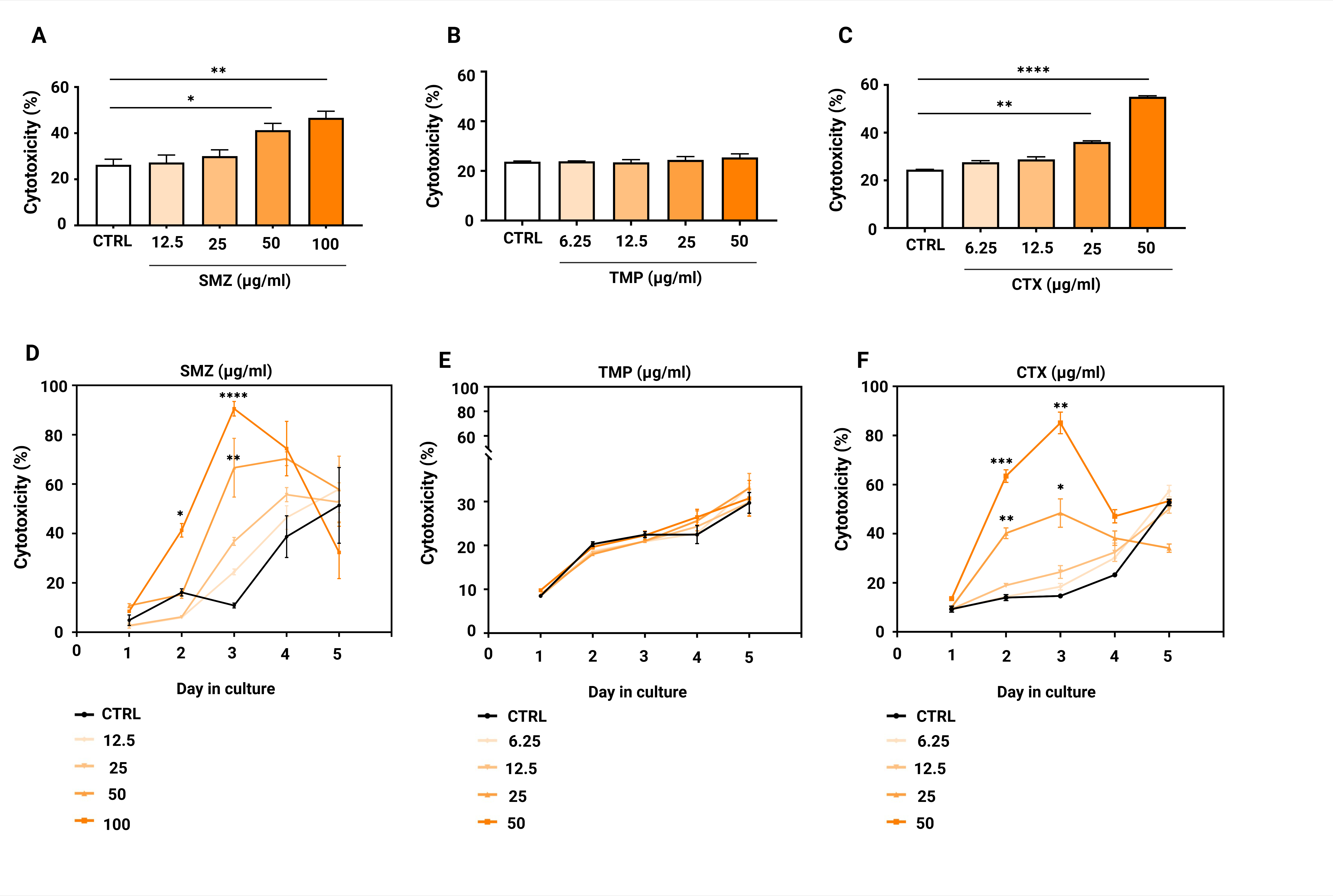


**Supplementary Figure 2. Evaluation of the chronic cytotoxic effects of SMZ, TMP, or CTX on BeWo cells over five days. (A) (B) (C),** Bar plots showing the cytotoxicity (%) of BeWo cells after 5 days exposure to varying concentrations of SMZ **(A)**, TMP **(B)**, and CTX **(C)**, as measured by LDH assay. Cytotoxicity significantly increased for SMZ at 50 µg/ml and 100 µg/ml and for CTX at 25 and 50 µg/ml compared to untreated (control) cells. Prolonged (5 days) TMP treatment did not result in significant cytotoxicity. **(D) (E) (F).** Line graphs representing time-course cytotoxicity over five days of culture with SMZ **(D)**, TMP **(E)**, and CTX **(F)**. SMZ and CTX exhibited time-dependent cytotoxicity, with significant effects from day 2 at 100 µg/ml SMZ and 50 µg/ml CTX, whereas TMP showed no substantial cytotoxic impact over time. All graphs represent three independent experiments (N=3), each including three technical repeats (n=3). Data were analyzed by one-way ANOVA and are presented as mean ± SEM. Statistical significance is indicated as: **p*<0.05, ***p*<0.01, ****p*<0.001, *****p*<0.0001. **SMZ**: Sulfamethoxazole, **TMP**: Trimethoprim, **CTX**: Co-trimoxazole, **CTRL**: Control.


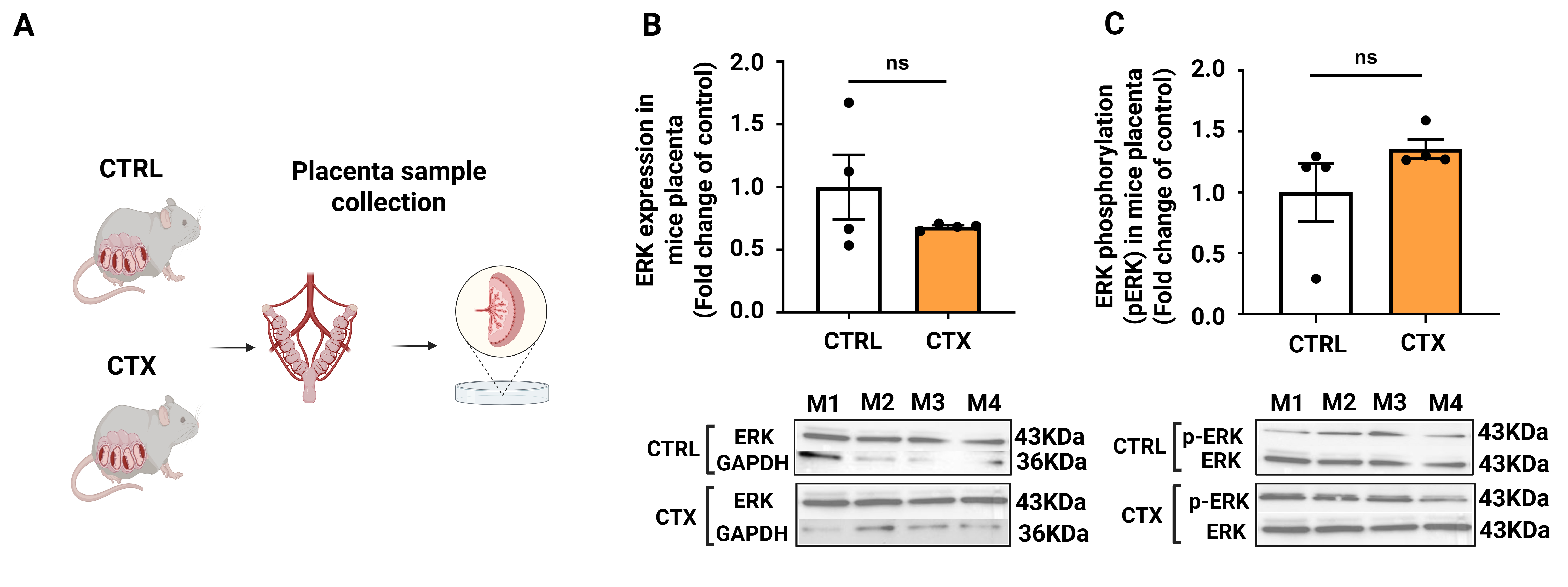


**Supplementary Figure 3. Analysis of ERK expression and phosphorylation in placental samples of CTX-exposed and control pregnant mice. (A)** Schematic representation of *in vivo* placenta sample collection from control (CTRL) and co-trimoxazole (CTX)-treated mice during pregnancy. **(B)** Comparison of total ERK protein expression in placental tissue between CTRL and CTX groups, normalized to GAPDH as the reference protein. Representative Western blot images are shown for four mice as replicates (M1-M4). **(C)** Comparison of phosphorylated ERK (p-ERK) levels, normalized to total ERK protein, in placental samples between CTRL and CTX groups. Corresponding Western blot images for p-ERK and total ERK are shown for each mouse. Statistical analysis by Student's t-test revealed no significant differences (ns) between groups in ERK expression or phosphorylation. Graphs B and C represent four mice for each experimental group (N=4). Data were analyzed by Student's t-test and are presented as mean ± SEM. **CTX**: Co-trimoxazole, **CTRL**: Control, **ERK**: Extracellular signal-regulated kinase, **p-ERK**: phosphorylated-ERK, **GAPDH**: Glyceraldehyde-3-phosphate dehydrogenase. **M1:** Mouse 1, **M2:** Mouse 2, **M3:** Mouse 3, **M4:** Mouse 4.


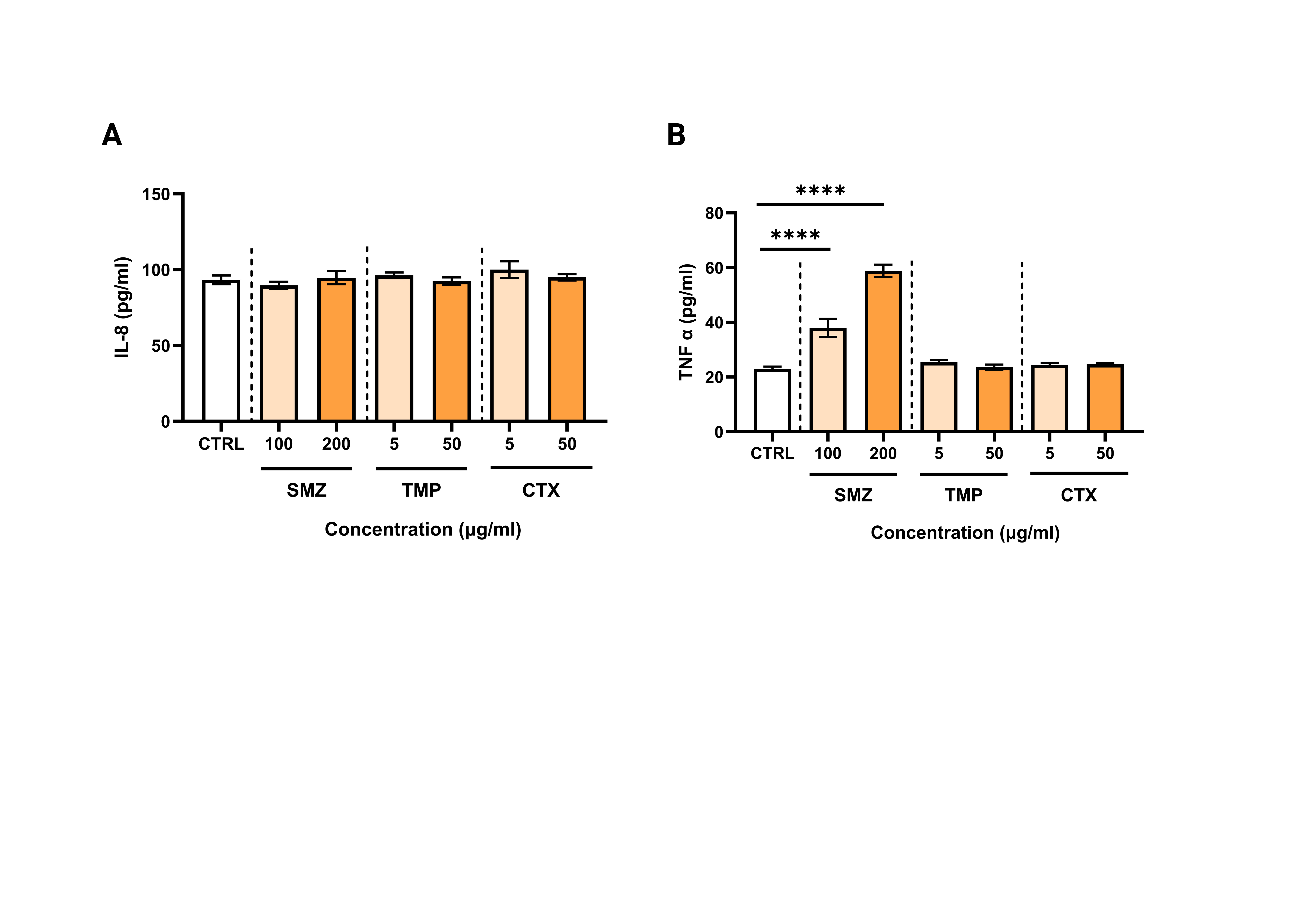


**Supplementary Figure 4. Effects of SMZ, TMP, and CTX on IL-8 and TNFα secretion from BeWo cells. (A)** IL-8 and **(B)** TNF-α concentrations in cell culture supernatants following exposure to SMZ, TMP, or CTX at the indicated concentrations (µg/mL). Cytokine levels were quantified by ELISA and are expressed as pg/mL. Both graphs represent three independent experiments (N=3), each including three pooled technical repeats (n=3). Data were analyzed by one-way ANOVA and are presented as mean ± SEM. Statistical significance is indicated as: **** *p*<0.0001 compared with the control group. **IL-8**: Interleukin-8, **TNFα**: Tumor Necrosis Factor alpha, **SMZ**: Sulfamethoxazole, **TMP**: Trimethoprim, **CTX**: Co-trimoxazole.

**Supplementary Table 1. Primers used for gene expression analysis.**

| **Gene** | **Sequences (5’-3’)** | | **Species** | **AT**  **(^°^C)** |
| --- | --- | --- | --- | --- |
|  | **Forward** | **Reverse** |  |  |
| *Β-actin* | CTGGAACGGTGAAGGTGACA | AAGGGACTTCCTGTAACAATGCA | Homo Sapiens | 63 |
| *IL-6* | TACCCCCAGGAGAAGATTCC | TTTTCTGCCAGTGCCTCTTT | Homo Sapiens | 63 |
| *RPL13A* | CCTGGAGGAGAAGAGGAAAGAGA | TTGAGGACCTCTGTGTATTTGTCAA | Mus musculus | 61.4 |
| *IL-6* | CTGCAAGAGACTTCCATCCAG | AGTGGTATAGACAGGTCTGTTGG | Mus musculus | 59 |
